# Plasmatic higher levels of homocysteine in Non-alcoholic fatty liver disease (NAFLD)

**DOI:** 10.1186/1475-2891-12-37

**Published:** 2013-04-02

**Authors:** Sylene Coutinho Rampche de Carvalho, Maria Tereza Cartaxo Muniz, Maria Deozete Vieira Siqueira, Erika Rabelo Forte Siqueira, Adriana Vieira Gomes, Karina Alves Silva, Laís Carvalho Luma Bezerra, Vânia D’Almeida, Claudia Pinto Marques Souza de Oliveira, Leila Maria M Beltrão Pereira

**Affiliations:** 1 School of Medicine, Universityof Pernambuco, Pernambuco, Brazil; 2 Pediatrics Hematology and Oncology Center, University of Pernambuco, Pernambuco, Brazil; 3 Liver Institute of Pernambuco, Pernambuco, Brazil; 4 Biological Science Institute, University of Pernambuco, Pernambuco, Brazil; 5 Department of Pediatrics, Federal University of São Paulo, São Paulo, Brazil; 6 School of Medicine, University of São Paulo, São Paulo, Brazil; 7 Instituto do Fígado de Pernambuco, Arnóbio Marques Street, 310, Santo Amaro- Recife, PE, Zip Code: 50.100-130, Brazil

**Keywords:** Fatty liver, Non-alcoholic steatohepatitis, Methylenetetrahydrofolate reductase (*MTHFR*), Oxidative stress, Polymorphisms

## Abstract

**Background:**

Non-alcoholic fatty liver disease (NAFLD) is a chronic liver disease, which includes a spectrum of hepatic pathology such as simple steatosis, steatohepatitis, fibrosis and cirrhosis. The increased serum levels of homocysteine (Hcy) may be associated with hepatic fat accumulation. Genetic mutations in the folate route may only mildly impair Hcy metabolism. The aim of this study was to investigate the relation between liver steatosis with plasma homocysteine level and MTHFR C677T and A1298C polymorphisms in Brazilian patients with NAFLD.

**Methods:**

Thirty-five patients diagnosed with NAFLD by liver biopsy and forty-five healthy controls neither age nor sex matched were genotyped for C677T and A1298C MTHFR polymorphisms using PCR-RFLP and PCR-ASA, respectively, and Hcy was determined by HPLC. All patients were negative for markers of Wilson’s, hemochromatosis and autoimmune diseases. Their daily alcohol intake was less than 100 g/week. A set of metabolic and serum lipid markers were also measured at the time of liver biopsies.

**Results:**

The plasma Hcy level was higher in NAFLD patients compared to the control group (p = 0.0341). No statistical difference for genotypes 677C/T (p = 0.110) and 1298A/C (p = 0.343) in patients with NAFLD and control subjects was observed. The genotypes distribution was in Hardy-Weinberg equilibrium (677C/T p = 0.694 and 1298 A/C p = 0.188). The group of patients and controls showed a statistically significant difference (p < 0.001) for BMI and HOMA_IR, similarly to HDL cholesterol levels (p < 0,006), AST, ALT, γGT, AP and triglycerides levels (p < 0.001). A negative correlation was observed between levels of vitamin B12 and Hcy concentration (p = 0.005).

**Conclusion:**

Our results indicate that plasma Hcy was higher in NAFLD than controls. The MTHFR C677T and A1298C polymorphisms did not differ significantly between groups, despite the 677TT homozygous frequency was higher in patients (17.14%) than in controls (677TT = 4.44%) (p > 0.05). The suggested genetic susceptibility to the MTHFR C677T and A1298C should be confirmed in large population based studies.

## Background

Nonalcoholic fatty liver disease (NAFLD) is a chronic liver disease, which presents a spectrum of hepatic pathology including simple steatosis, steatohepatitis (NASH), fibrosis, and cirrhosis [1]. NALFD is now the most common cause of abnormal liver biochemistry in North America [2] and is also known to be associated with some drugs, genetic defects, obesity, insulin resistance and type 2 diabetes [3]. The accumulation of triglycerides in the liver in the absence of excess alcohol intake has been described in the early sixties [4] and predominantly characterized by macrovesicular hepatic steatosis [5].

The two metabolic abnormalities mostly associated with NAFLD are insulin resistance (IR) and an increased supply of fatty acids to the liver [6,7]. As adipose tissue becomes resistant to insulin, serum lipoprotein levels shift and flux of free fatty acids to the liver increases [8,9]. The cumulative effects of insulin resistance and increased circulating free fatty acids act in concert to channel fatty acids into storage rather than into secretory and pathways of degradation [10,11]. Therefore, it is mainly associated with other clinical expressions of IR, such as metabolic syndrome and its features, as obesity, type 2 diabetes, dyslipidemia and hypertension [12].

In addition, it has been reported that Hyperhomocysteinemia (HHcy) alters intracellular lipid metabolism [13]. Thus the data support the view that increased serum levels of homocysteine (Hcy) may be associated with hepatic fat accumulation. Homocysteine is a sulphur-containing amino acid, which is an intermediate product in the normal biosynthesis of the amino acids methionine and cysteine [14].

Some genetic mutations in the folate route may mildly impair homocysteine metabolism [4,15]. The genomic DNA methylation directly correlates with folate status and inversely, with plasma homocysteine levels. The methylenetetrahydrofolate reductase (*MTHFR*) polymorphisms influence DNA methylation status through an interaction with folate status [15]. Mutations in *MTHFR* gene (C677T and A1298C) result in amino acids substitutions that lead to a decreased enzyme activity, reducing the 5 mTHF availability [16]. The *MTHFR* C677T and A1298C polymorphisms have been shown to be associated with higher levels of homocysteine, when plasma folate levels are low [16,17].

Several studies have been conducted in order to find a relationship between the presence of *MTHFR* polymorphisms and disease risk. The C677T and A1298C polymorphisms affect a large portion of the population with considerable variations between different ethnic groups [18]. Although Brazil has become the object of interest in population genetic studies because of phenotypic and social differences observed among populations from five geographic regions of the country, studies with *MTHFR* C677T and A1298C polymorphisms in Brazilian population are necessary, especially when associated with NAFLD. Therefore, the aim of this study was to investigate the relation between liver steatosis with plasma homocysteine levels and *MTHFR* C677T and A1298C polymorphisms in patients with NAFLD from Northeast Brazil.

## Methods

This study comprised 35 patients with a diagnosis of NAFLD based on liver biopsy findings (09 males and 26 females, age mean 49 years) and 51 healthy subjects, without NAFLD (16 males and 35 females, age mean 39 years), according to ultrasound findings at the Liver Institute of Pernambuco – Brazil between 2005 to 2008. In addition, all patients had elevated alanine aminotransferase (ALT) and/or aspartate aminotransferase (AST) levels at least on two occasions, over 6 months prior to enrollment. The study protocol was approved by the Ethics Committee for Human Hesearch of the University of Pernambuco and a written consent was obtained from every individual participating in the study. This transversal study was conducted in accordance with the Helsinki declaration of 2008.

All patients were negative for markers of Wilson’s disease, hemochromatosis and autoimmune diseases and had current and past daily alcohol intake kept under 100 g/week. Patients who were hepatitis B surface antigen– and/or HIV-positive and had other potential causes of liver disease were excluded. Patients with clinically decompensated cirrhosis or contraindications for liver biopsy were not included in the study.

None of the patients were taking medication that could cause steatosis (salicylates, nonsteroidal anti-inflammatory drugs, corticosteroids, valproic acid, amiodarone, perhexiline maleate) or modify serum levels of homocysteinemia (folate, vitamin B12).

Diagnosis of type 2 diabetes and dyslipidemia were based on the criteria of the American Diabetes Association (fasting glucose ≥ 100 mg/dL; Triglyceride ≥ 150 mg/dL HDL < 40 mg/dl in man or < 50 mg/dL in woman) [19]. Overweight corresponded to Body Mass Index (BMI) > 25 kg/m^2^ and obesity to BMI ≥ 30 kg/m^2^.

### Laboratory assays

Blood samples were collected after fasting overnight and centrifuged within 60 min to separate plasma, serum and leukocyte cells and storaged at – 80°C.

Fasting Glucose, total cholesterol and fractions, triglycerides, alanine aminotransferase (ALT), aspartate aminotransferase (AST), alkaline phosphatase (AP), γGT were performed by standard methods using automated techniques (Modular P800, Hitach/Roche) in all patients at basal line and at the end of the study.

The homocysteine levels were determined by HPLC (high performance liquid cromatography) with fluorimetric detection [20]. The folic acid and B12 vitamin were determined by standard methods using automated techniques (Elecsys and COBAS analyzers/Roche).

The insulin resistance index was calculated based on fasting insulin and fasting glucose according to homeostasis model assessment (HOMA -IR) [21]. The Body Mass Index (BMI) is defined as the individual’s body mass divided by the square of his or her height.

For *MTHFR* polymorphism identification, the DNA was extracted from leukocytes by the salting out method*.* The C677T and A1298C *MTHFR* polymorphisms were determined by PCR-RFLP (*Hinf* I) and PCR-ASA, respectively [22,23]. The amplified and digested fragments were analyzed in 3% agarose gel and the fragments were visualized in ultraviolet light (UV) after being stained with ethidium bromide. The 677 wild type (CC) shows a single fragment of 198 bp; heterozygote (CT) shows fragments of 198, 175 and 23 bp; and mutant homozygote (TT) shows two fragments with 175 and 23 bp [22]. The polymorphism *MTHFR* A1298C wild type and mutated alleles yield fragments of 77-bp and 120-bp, respectively [23].

### Histological analysis

A single liver pathologist scored all specimens with expertise in NAFLD: macro and microvacuolar fatty change, zonal distribution, foci of necrosis, portal and perivenular fibrosis, inflammatory and fibrotic infiltrate with zonal distribution. Macrovesicular steatosis was classified in low steatosis (<33% of hepatocytes with steatosis), moderate (34-66%) and intense (>66%) [24].

### Statistical analysis

Data analysis was performed with BioEstat 5.0 software. The quantitative variables were described by mean values ± SD. *T*-test and Mann–Whitney *U* test were used in variables with normal and without normal distribution. Spearman’s *r* coefficient was used to discover a correlation between continuous variables (folate and B12 vitamin status and homocysteine). The frequencies of each allele were calculated as q = (2a + b)/n, where **a** corresponded to the number of homozygotes, **b** to the number of heterozygotes, and **n** to the number of alleles analyzed, respectively. Hardy-Weinberg equilibrium was tested for the SNP by comparing observed frequencies with expected frequencies and using a *χ*2 test. The differences in genotypes from each polymorphic position between cases and controls were assessed by Fisher’s exact tests. In all statistical evaluations, *P* < 0.05 was taken as significant.

## Results

### Clinical and biochemical analysis

The results of the clinical and biochemistry parameters are described in Table 1. Thirty-five patients had a clinical and biochemical analysis completed in the study. There were 25.7% (6/35) males and 74.3% (26/35) females. The BMI and HOMA -IR were higher in NAFLD patients than in control groups (p < 0.001). Similarly to HDL cholesterol levels (p < 0,006), the AST, ALT, γGT, AP and triglycerides levels differed significantly in NAFLD patients as compared to controls (p < 0.001).

**Table 1:** Clinical and biochemical characteristics in NAFLD patients and controls subjects

	**PATIENTS (n=35)**	**CONTROLS (n=51)**	***P***
Total Cholesterol	187.91 ± 38.83	182.27 ± 38.36	0.516
HDL	44.30 ± 13.83	53.51 ± 15.27	0.006*
LDL	115.49 ± 35.88	110.46 ± 32.38	0.509
Triglycerides	171.72 ± 80.91	93.77 ± 39.70	p< 0.001*
Fasting Glucose	99.67 ± 35.59	92.09 ± 10.50	0.167
Insulin	16.55 ± 10.74	7.12 ± 4.22	p < 0.001*
HOMA value >3.0	3.90 ± 2.75	1.65 ± 1.10	p < 0.001*
AST	54.51 ± 31.94	18.20 ± 5.93	p< 0.001*
ALT	82.15 ± 38.72	14.82 ± 8.99	p < 0.001*
GGT	126.38 ± 125.90	28.35 ± 22.61	p< 0.001*
AP	87.15 ± 53.88	63.92 ± 33.53	0.019*
BMI	29.77 ± 4.38	24.22 ± 3.68	p < 0.001*

* t-Test was applied for these groups specifically. *BMI*. body mass index; *HDL-C*. high-density-lipoprotein cholesterol; *LDL-C*. Low-density- lipoprotein cholesterol.

Table 2 shows the results of B12 vitamin levels demonstrating a significant difference between patients and controls. In addition, when comparing NALFD patients with controls as to Hcy levels, a significant difference between these groups was shown (p = *0.0341*).

**Table 2:** Relationship among homocysteine, folate and B12 vitamin in NAFLD patients and in control subjects

	**PATIENTS (n=35)**	**CONTROLS (n=51)**	***P***
Folate	15.25 ± 3.27	15.12 ± 3.02	0.853
B12 vitamin	473.11 ± 199.40	355.02 ± 178.04	0.005
Homocystein	9.69 ± 2.89	8.49 ± 1.76	0.034

p = t-Test was applied for these groups specifically.

### Polymorphisms analysis

The *MTHFR* polymorphisms were analyzed from peripheral blood of 35 patients and 45 controls. The frequencies of the *MTHFR* genotypes for both loci C677T and A1298C and respective alleles are shown in Table 3. The distributions of the *MTHFR* genotypes correspond to those expected by Hardy-Weinberg equilibrium in both NAFLD patients and controls indicating that the allelic distribution was random. The genotypes CT (48.57%) and AA (57.15%) were more frequent in NAFLD patients for C677T and A1298C, respectively. Although the 677TT homozygous frequency was higher in patients (17.14%) than in controls (677TT = 4.44%), as expected, the difference in genotypes distribution was not significant (p = 0.110). No statistical differences were observed in A1298C genotypes and alleles, either (p = 0.343). No differences in the C677T (*p* = 0.110) and A1298C (*p* = 0.343) *MTHFR* polymorphisms distributions were found between patients and controls (Table 3).

**Table 3:** **Genotypes and alleles frequencies of the C667T and A1298C ( *MTHFR *) polymorphisms in NAFLD patients and control subjects and statistical parameters**

**Genotypes and alleles**	**NAFLD N (35) %**	P^*^	**Control N (45)%**	P^**^	**χ**^**2**^	P^***^
*MTHFR* C677T						
CC	12 (34.29)	0.996	23 (51.11)	0.360	0.154	0.345
CT	17 (48.57)		20 (44.45)			
TT	06 (17.14)		02 (4.44)			
CT+TT	23 (63.71)		22 (36.30)			
Allele 677C	21 (0.58)		33 (0.73)			
Allele 677T	14 (0.41)		12 (0.26)			
*MTHFR* A1298C						
AA	20 (57.15)	0.106	26 (57.79)	0.707	1.727	0.473
AC	15 (42.85)		17 (37.77)			
CC	0		02 (4.44)			
AC+CC	15 (50.31)		19 (49.68)			
Allele 1298A	24 (0.78)		35 (0.76)			
Allele 1298C	4 (0.21)		10 (0.23)			

P^*^= Hardy-Weinberg Equilibrium *NAFLD*; P^*^= Hardy-Weinberg Equilibrium Control; χ^2^ = chi-square; P^***^ = Exact Fisher.

## Discussion

We designed our study based on the hypothesis that the homozygosity for both polymorphisms, C677T and A1298C, significantly raises the levels of plasma Hcy. However, the *MTHFR* C677T and A1298C polymorphisms did not differ significantly between groups in this study. This fact can be explained by two reasons, (1) the sample size is small and (2) the mixing occurred in the region, since the northeast Brazilian population was originated from African, Caucasian and Native American ancestral individuals [25]. According to Volcik et al. (2001) the frequencies of alleles 677 T and A1298C may vary according to geographical area and ethnic group and the difference of values observed among populations can be explained by ethnic differences and nutrition [26]. Our results indicated that, despite the small number of northeastern Brazilian patients with NAFLD in our sample, NAFLD was associated with elevated plasma Hcy.

Association studies of *MTHFR* gene polymorphisms and NAFLD disease, such as those of Serin (2006) and Sazci (2008) cited, are scarce. Both studies were developed with the Turkish population [3,27]. Our study is the first description of C677T and A1298T *MTHFR* polymorphism in a sample of northeastern Brazilians with NAFLD.

In this study there was a statistically significant difference for BMI and HOMA_IR between groups of patients and controls, but there was no correlation between homocysteine concentration and the other variables studied in patients with NAFLD, except the negative correlation observed between levels of vitamin B12 and homocysteine concentration (p = 0.006). These results are consistent with Gulsen et al. that also found a negative correlation between homocysteine and B12 [28], probably because of the lower intake of essential vitamins such as folate and vitamin B12 in these patients with NAFLD. Hcy can result from deficiencies of vitamin cofactors (B6, B12, folic acid) required for Hcy metabolism and/or from genetic disorders of its metabolism [29]. These data support the view that increased serum levels of homocysteine may be associated with hepatic fat accumulation. Moreover, the BMI and HOMA -IR were higher in NAFLD patients and also the relationship between Hcy and B12 vitamin was significant between NAFLD and control group. The triglycerides levels and HDL cholesterol were significantly different in NAFLD patients compared to controls. Siqueira et al. (2011) related that plasma Hcy levels is highly prevalent in subjects with chronic hepatits C with steatosis regardless of HCV genotype and vitamin deficiency [30].

The present study shows that the plasma Hcy was higher in patients with NAFLD than in healthy subjects, but this study does not allow any conclusion as to whether the increase of plasma Hcy is the cause of insulin resistance and whether the plasma Hcy concentrations correlates with the stage of the disease in NAFLD.

## Conclusion

In conclusion, the results indicated that in patients from Northeast Brazil, NAFLD is associated with elevated plasma Hcy. NAFLD, apparently, was associated with other known host features such as BMI, HOMA, and levels of serum lipids. Further studies with larger samples need be conducted to confirm or exclude the relations found herein, as well as analyses of the *MTHFR* C677T and A1298C polymorphism frequencies.

## Abbreviations

MTHFR: Methylenetetrahydrofolate reductase; NAFLD: Nonalcoholic fatty liver disease; HHcy: Hyperhomocysteinemia; Hcy: Homocysteine; PCR-RFLP: Polymerase chain reaction - restriction fragment length polymorphism; PCR-ASA: Polymerase chain reaction - amplicon sequence analysis; HPLC: High performance liquid cromatography; BMI: Body mass index; HOMA_IR: Homeostasis model assessment _ insulin resistance; NASH: Nonalcoholic steatohepatitis; IR: Insulin resistance; ALT: Alanine aminotransferase; AST: Aspartate aminotransferase; AP: Alkaline phosphatase; HDL: High density lipoprotein; ROC: Receiver operating characteristic; UV: Ultraviolet light; SNP: Single nucleotide polymorphism; γGT: γ-glutamyltransferase; SREBP: Sterol regulatory element-binding proteins

## Competing interests

The authors declare that they have no competing interests.

## Authors’ contributions

SCRC participated in the all steps of study, including design of the study, performed the statistical analysis and wrote the manuscript. MTCM participated in the sequence alignment and drafted the manuscript. MDVS carried out the molecular genetic studies of A1298C polymorphism. ERFS critical revision of the manuscript for important intellectual content. AVG critical revision of the manuscript for important intellectual content. KAS acquisition of data, analysis and interpretation of data. LCLB carried out the molecular genetic studies of C677T polymorphism. VDA measured the plasma Hcy level in this study. CPMS helped to draft the manuscript. LMMBP conceived of the study and participated in its design and coordination. All authors read and approved the final manuscript.
